# Effect of caudal ketamine on minimum local anesthetic concentration of ropivacaine in children: a prospective randomized trial

**DOI:** 10.1186/s12871-020-01058-y

**Published:** 2020-06-08

**Authors:** Huai-Zhen Wang, Ling-Yu Wang, Hui-Hong Liang, Yan-Ting Fan, Xing-Rong Song, Ying-Jun She

**Affiliations:** Department of Anesthesiology and Perioperative Medicine, Guangzhou Women and Children’s Medical Center, Guangzhou Medical University, 9# Jinsui Road, Guangzhou, 510623 China

**Keywords:** Anesthesia, caudal, Ketamine, Ropivacaine, Analgesia, Anesthetic techniques

## Abstract

**Background:**

Caudal ketamine has been shown to provide an effective and prolonged post-operative analgesia with few adverse effects. However, the effect of caudal ketamine on the minimum local anesthetic concentration (MLAC) of ropivacaine for intra-operative analgesia is unclear.

**Methods:**

One hundred and sixty-nine children were randomized to five groups: Group C (caudal ropivacaine only), Group K_0.25_ (caudal ropivacaine plus 0.25 mg/kg ketamine), Group K_0.5_ (caudal ropivacaine plus 0.5 mg/kg ketamine), Group K_0.75_ (caudal ropivacaine plus 0.75 mg/kg ketamine), and Group K_1.0_ (caudal ropivacaine plus 1.0 mg/kg ketamine). The primary outcome was the MLAC values of ropivacaine with/without ketamine for caudal block.

**Results:**

The MLAC values of ropivacaine were 0.128% (0.028%) in the control group, 0.112% (0.021%) in Group K_0.25_, 0.112% (0.018%) in Group K_0.5_, 0.110% (0.019%) in Group K_0.75_, and 0.110% (0.020%) in Group K_1.0_. There were no significant differences among the five groups for the MLAC values (*p* = 0.11). During the post-operative period the mean durations of analgesia were 270, 381, 430, 494, and 591 min in the control, K_0.25_, K_0. 5_, K_0.75_, and K_1.0_ groups respectively, which shown that control group is significantly different from all ketamine groups. Also there were significant differences between K_0.25_ and K_0.75_ groups, and between K_1.0_ groups and the other ketamine groups.

**Conclusions:**

Adding caudal ketamine to ropivacaine prolong the duration of post-operative analgesia; however, it does not decrease the MLAC of caudal ropivacaine for intra-operative analgesia in children.

**Clinical trial registration:**

ChiCTR-TRC-13003492. Registered on 13 August 2013.

## Background

A caudal block is commonly used in children during lower abdominal and limb surgeries because it provides safe and effective perioperative analgesia. Single dose caudal levobupivacaine or ropivacaine provides analgesia for 4–8 h and has gained popularity with a high success rate and a low incidence of adverse effects [1, 2]. Also, it has been proved that several additives with local anesthetics can prolong the duration of caudal analgesia [3]. Research discovered that the most commonly used additives were clonidine (42.3%), ketamine (37.5%), opioids (18.1%), adrenaline (1.8%), and midazolam (0.3%) [4].

Caudal block is usually injected by a single high-dose local anesthetic, while decreasing local anesthetic dose reduces local tissue and systemic toxicity. Adjuvants were usually used to decrease the dose of local anesthetic and prolong the duration of analgesia. The minimum local anesthetic concentration (MLAC) has been conventionally used for evaluating the relative efficacy of generally used local anesthetics [5–7]. Many additives have been administrated in order to decrease the concentration of local anesthetics for caudal analgesia, while the effect of some additives on the MLAC values of local anesthetics have been explored in some previous studies [8, 9]. Caudal clonidine and dexmedetomidine produced a local anesthetic sparing effect with a dose-dependent decrease in MLAC of levobupivacaine for caudal anesthesia [8, 9]. As a commonly used additive, caudal ketamine has been shown to provide an effective and prolonged post-operative analgesia with few adverse events. However, the effect of caudal ketamine on the MLAC of ropivacaine is unclear. The aim of this prospective, randomized, and double-blind trial was to observe whether ketamine reduces the MLAC of ropivacaine for caudal block in children.

## Methods

This study was approved by the Institutional Review Board of Guangzhou Women and Children’s Medical Center (IRB2013053103), and written informed patient consent was attained from the parents or legal guardians of each paediatric patient in this trial. The trial was registered prior to patient enrolment at chictr.org.cn (ChiCTR-TRC-13003492). This study adhered to CONSORT guidelines for randomized trials. One hundred and sixty nine ASA physical status I or II children aged from 1 to 3 years old scheduled to undergo elective hydrocele or inguinal hernia repair were recruited. Exclusion criteria included patients with neurological, psychiatric, cardiovascular, respiratory, hepatic, renal diseases or bleeding disorders; or a known allergy history of anesthetics. Using a computer-generated table and a sealed envelope with sequence of numbers, children were randomly allocated into five groups into one of the five groups: Group C (caudal ropivacaine only), Group K_0.25_ (caudal ropivacaine plus 0.25 mg/ml preservative free ketamine), Group K_0.5_ (caudal ropivacaine plus 0.5 mg/ml preservative free ketamine), Group K_0.75_ (caudal ropivacaine plus 0.75 mg/ml preservative free ketamine), and Group K_1.0_ (caudal ropivacaine plus 1.0 mg/ml preservative free ketamine). The total volume of ropivacaine with/without preservative free ketamine solution for caudal injection in all groups are 1 ml/kg.

All children underwent preoperative fasting for 6 h and received no premedication. Sevoflurane via a face mask was used for anesthesia induction in the children. After securing intravenous access, 3 mg/kg propofol was intravenously administered to maintain sedation in a single dose or repeated doses. A caudal puncture was performed with a 22-gauge catheter by introducing in the caudal space with the patient in the left lateral decubitus position by a consultant anesthesiologist. The mixed solution of caudal injection is prepared by 0.9% saline, ropivacaine and ketamine. After felling the loss of resistance and checking for the negative presence of cerebrospinal fluid or blood, the ropivacaine with/without preservative free ketamine solution (total volume, 1 ml/kg) was administered via the catheter over 60 s. All inhalational agents were discontinued after the finish of the caudal injection. Then the patient was repositioned to supine for surgery. Continuously intravenous infused propofol (8–10 mg/kg/h) was administered to maintain sedation, and the depth of anesthesia was adjusted accordingly with the objective of achieving 80–120% baseline non-invasive mean arterial pressure. Dermatomal heights or upper limits of the caudal block were not formally assessed by pinprick before skin incision which was performed at exactly 20 min after the caudal injection. Baseline vital signs were collected to ensure a stable state and to discriminate slow onset of analgesia which was regarded as a confounding variable. An anesthesiologist who was unaware of the group assignments and the test concentrations of ropivacaine or ketamine maintain intraoperative depth of anesthesia, evaluated analgesic efficacy and collected the data. After skin incision, the patient was observed for at least 60 s for hemodynamic stability and signs of purposeful movement.

The primary outcome was the MLAC of ropivacaine with/without ketamine for caudal block in children. The success of a caudal block was defined as the change in hemodynamic parameters < 20% of baseline values and the absence of gross purposeful movement after skin incision. The modified Dixon’s up-and-down method was used to determine the target concentration of caudal ropivacaine for each pediatric patient, starting from 0.16% in each group, with 0.02% as a step size. According to the response of the previous patient in the same group, the target concentration of caudal ropivacaine was increased or decreased. If it failed, a 0.02% concentration of caudal ropivacaine would be increased for the next patient. If it was successful, the next child could be given caudal ropivacaine where the concentration would be decreased by 0.02%. When a response was determined to be a failure, the surgeon would stop all operations, and a rescue dose of 0.5 mg/kg ketamine would be intravenously administrated immediately to reduce the pain and could be repeated if needed during the surgery. A pair of crossover was defined as the midpoint between failed and successful caudal block in two consecutive children. And the study would be ended after collecting nine pairs of crossover in each group.

The secondary outcomes of the study were durations of analgesia. Postoperative analgesia was assessed by using the “Face, Legs, Activity, Cry, Consoling” tool (FLACC). A FLACC score of more than 4 points was considered as inadequate analgesia and was additional orally administrated 10 mg/kg ibuprofen every 6 h as needed. The duration time of analgesia was defined from caudal block to the first postoperative rescue analgesia. The independent nurses evaluated the pain scale every hour in 8 h after the surgery and then every 6 h as required. Beside these time points, the independent nurses will be on call to assess the pain scale of patients. Side effects like postoperative nausea and vomiting (PONV), urine retention, agitation, hypotension, bradycardia, and respiratory depression were recorded. Emergence agitation evaluation was scored by using a five-point scale. A score of ≥3 was defined as emergence agitation according to five-point scale (0 = falling asleep peacefully; 1 = quiet; 2 = easy to console; 3 = difficult to console with moderate agitation; 4 = combative, excited, and disorientated). On the second day after surgery all children were discharged.

### Statistical analysis

Statistical analyses were performed in SPSS 19.0 for Windows (SPSS Inc., USA). Data were expressed as mean (SD) or count, when appropriate. The MLAC was estimated from the up-and-down sequences with the Dixon and Massey method. The MLAC of caudal ropivacaine was analysed by calculating the midpoint concentration of all independent pairs of crossover points in each group. According to the study conducted by Paul and Fisher [10], nine pairs of crossover patients were needed. The variance of age, weight, duration of surgery, propofol doses, recovery time and duration of analgesia was analysed by using analysis of variance followed by a Dunnett t test or Kruskal-Wallis test. A Dunnett *t* test was used to test for normal distribution of the data. The duration of caudal analgesia was also analysed with Kaplan-Meier survival analysis and log-rank test. The type of surgery and adverse effects were analysed with the Chi-square test. *P* < 0.05 was considered statistically significant.

## Results

**Fig. 1:**
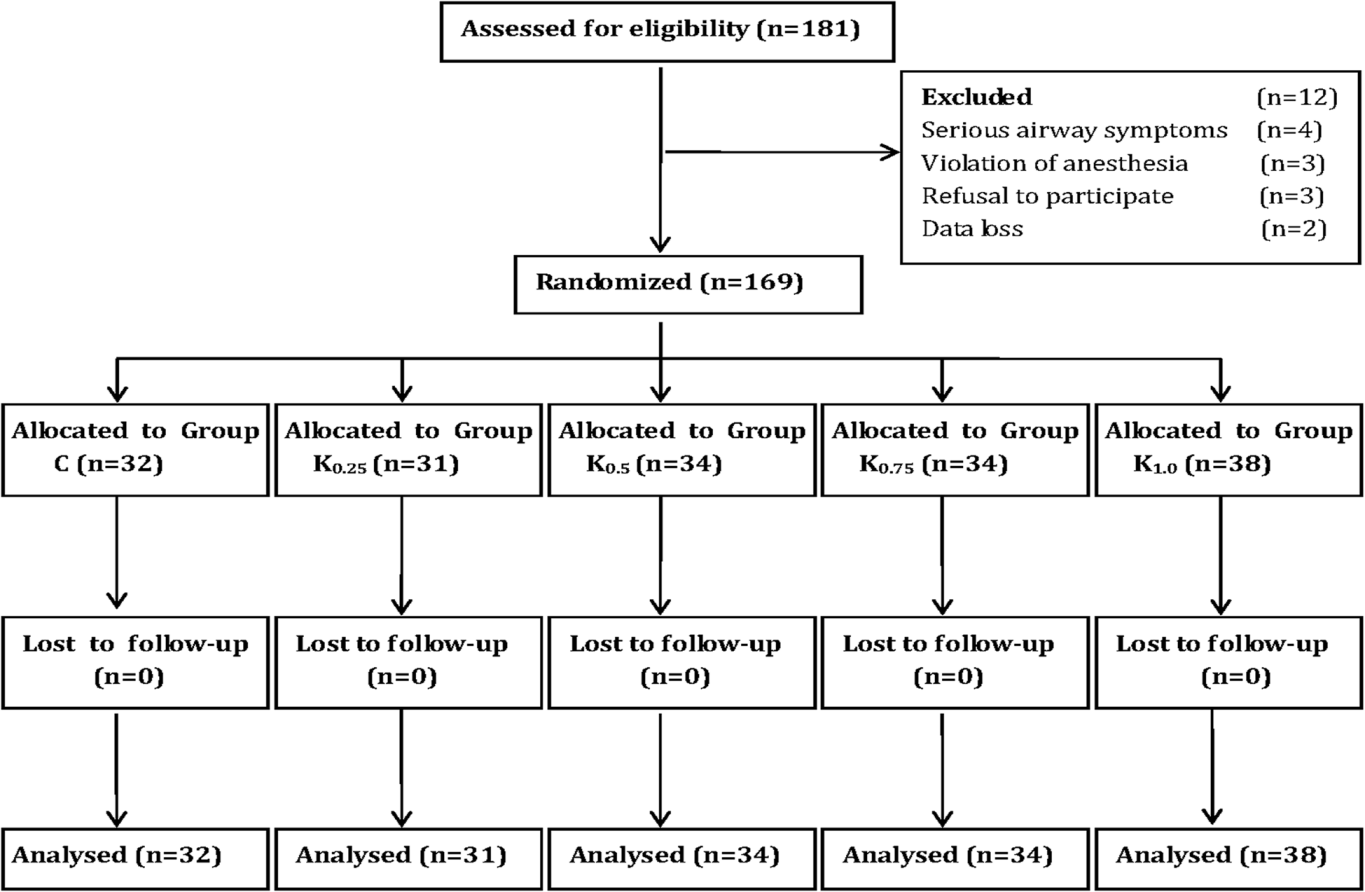
Consort flow diagram

One hundred and eighty-one children were enrolled in this study, 169 were analysed and 12 were excluded. Among the excluded patients, four were excluded because of serious airway symptoms, two were excluded because of data loss, three declined to participate in the study, and three were excluded due to the violation of anesthesia. All caudal block were considered as successful. The CONSORT flow diagram shows the details of patient recruitment (Fig. 1). Patient characteristics, the duration of operation, and the type of surgery were shown in Table 1. There were no significantly difference in incidences of agitation, urine retention, respiratory depression, bradycardia, hypotension, and PONV among groups (Table 1).

**Table 1:** Children’s demographic and experimental data and postoperative side effects data

Group	Group C	Group K_0.25_	Group K_0. 5_	Group K_0.75_	Group K_1.0_	P
Number of Patients (n)	32	31	34	34	38	/
Age (months)	22 (7)	20 (7)	21 (7)	19 (6)	20 (7)	0.653
Weight (kg)	11.6 (1.6)	11.3 (1.6)	11.8 (1.7)	11.4 (1.7)	11.3 (1.8)	0.737
Duration of surgery (min)	32 (9)	30 (7)	29 (5)	29 (6)	30 (8)	0.378
Recovery time (min)	31 (4)	31 (8)	31 (3)	30 (6)	30 (7)	0.850
Propofol dose (mg)	105 (27)	104 (26)	107 (30)	103 (28)	102 (28)	0.957
Type of surgery						
Inguinal hernia (n)	22	23	24	26	27	0.96
Hydrocele (n)	10	8	10	8	11	
PONV (n)	4	4	5	6	10	0.513
Agitation (n)	0	0	0	0	0	/
Urine retention (n)	1	0	0	0	0	0.366
Bradycardia (n)	1	1	2	1	3	0.822
Hypotension (n)	0	1	0	1	0	0.517
Respiratory depression (n)	0	0	0	0	0	/

Values are mean (SD) or numbers, Data are mean ± SD or numbers of cases (n)

**Fig. 2:**
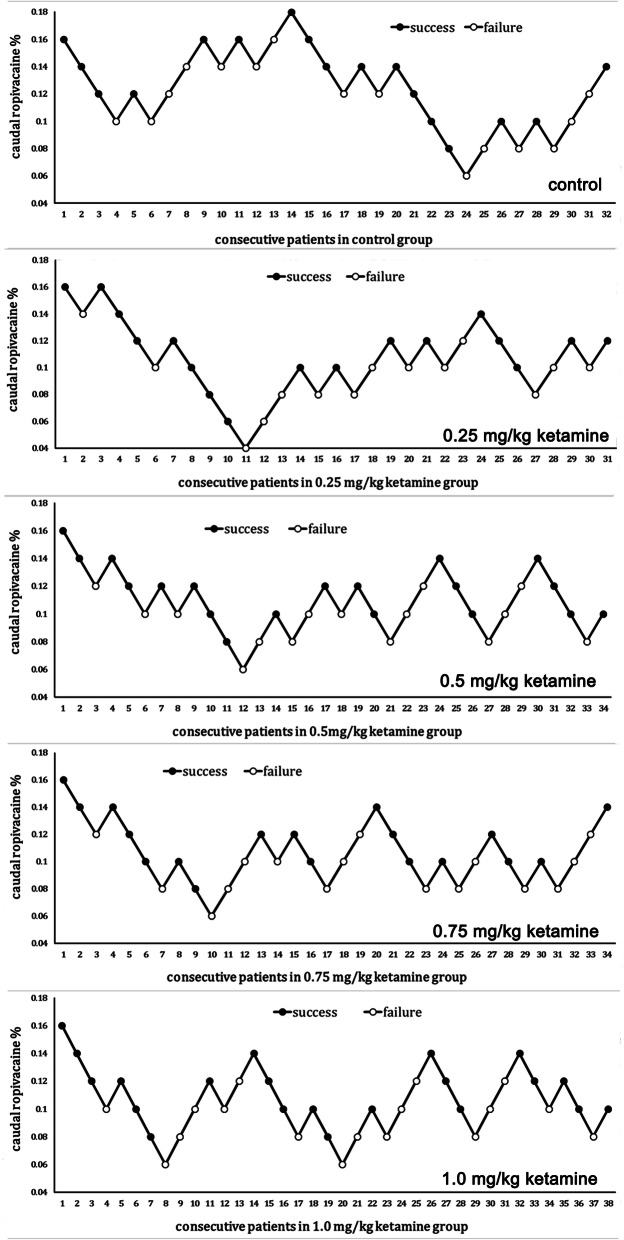
The response curve of consecutive children in each of the five groups. Skin incisions were attempted at different concentrations of caudal ropivacaine. The MLAC values of ropivacaine were 0.128% (0.03%) in the control group, 0.112% (0.02%) in Group K_0.25_, 0.112% (0.02%) in Group K_0.5_, 0.110% (0.02%) in Group K_0.75_, and 0.110% (0.02%) in Group K_1.0_. There were no significant differences among the five groups for the MLAC values (*p* = 0.11)

The patient responses to the skin incision according to the up-and-down sequence were illustrated in Fig. 2. The MLAC values of ropivacaine were 0.128% (0.028%) in control group, 0.112% (0.021%) in Group K_0.25_, 0.112% (0.018%) in Group K_0.5_, 0.110% (0.019%) in Group K_0.75_, and 0.110% (0.020%) in Group K_1.0_. There were no significant differences of the MLAC values among the five groups (*p* = 0.11).

**Fig. 3:**
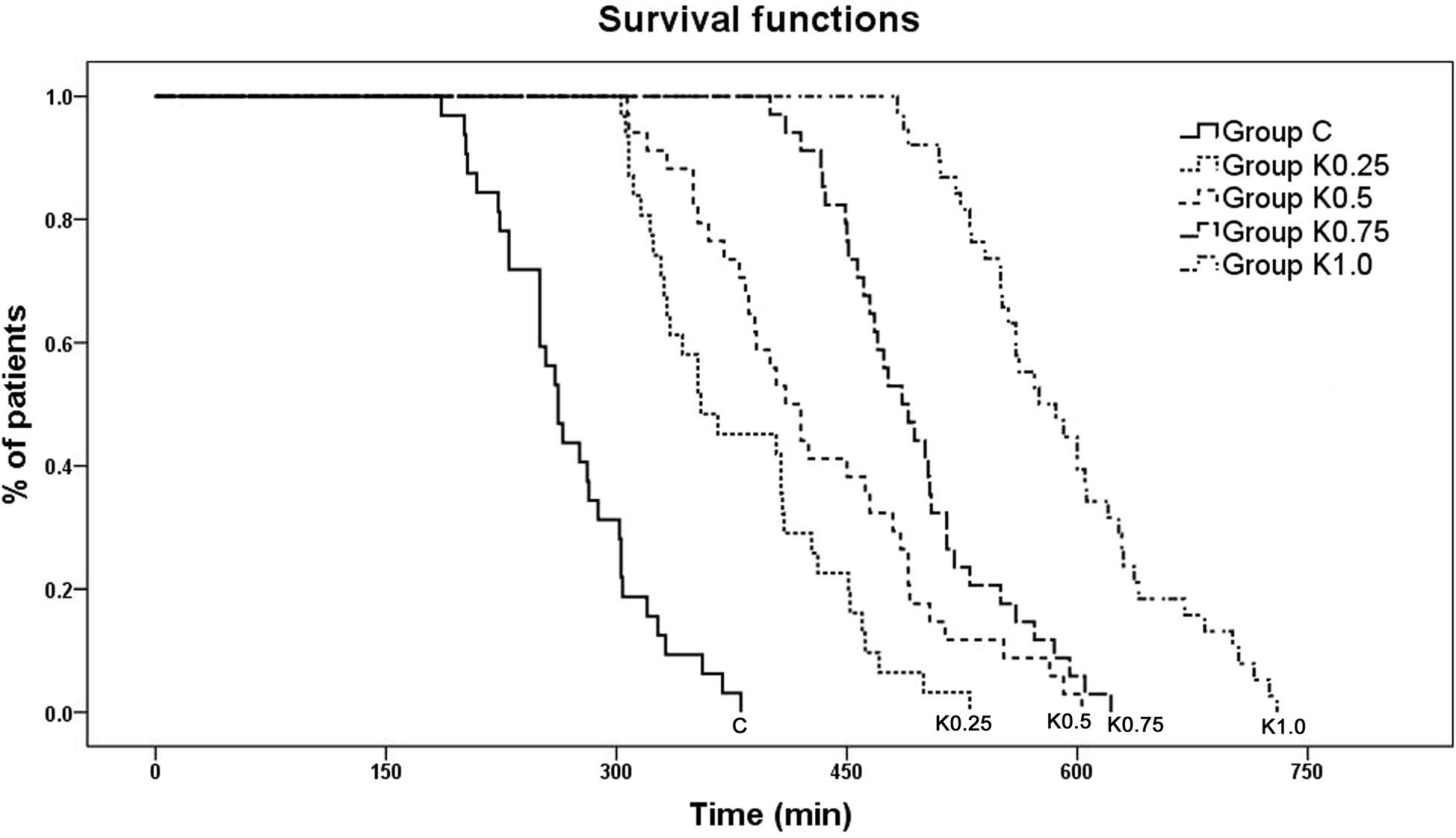
Results of Kaplan-Meier survival analysis were displayed as survival curves for the five caudal solution. The log-rank test was used to compare the rate of requirement for additional analgesia in the study

The durations of analgesia in the five groups were illustrated with Kaplan-Meier survival curves in Fig. 3. The mean durations of analgesia in the postoperative period were 270, 381, 430, 494, and 591 min in the control, K_0.25_, K_0.5_, K_0.75_, and K_1.0_ groups, respectively. There were significant differences between the control group and all ketamine groups (control VS K_0.25_, *p* = 0.01; control VS K_0.5_, control VS K_0.75_, control VS K_1.0_, *p* < 0.001), and also between K_1.0_ groups and the other ketamine groups (K_0.25_ VS K_1.0_, and K_0. 5_ VS K_1.0_, *p* < 0.001; K_0.75_ VS K_1.0_, *p* = 0.022). Significant differences were observed between K_0.25_ and K_0.75_ groups (*p* = 0.001), but not between K_0.25_ and K_0.5_ groups (*p* = 0.961), or between K_0.5_ and K_0.75_ groups (*p* = 0.222).

## Discussion

The aim of this study was to demonstrate whether caudal ketamine reduces the MLAC of caudal ropivacaine and prolongs the duration of post-operative analgesia in children. Our results showed that combined ketamine with ropivacaine for caudal block would prolong the duration of post-operative analgesia. However, caudal ketamine did not reduce the MLAC of caudal ropivacaine for intra-operative analgesia in children.

Some studies have determined the MLAC value of ropivacaine for caudal blocks in young children. Ingelmo et al. [7] investigated the MLAC of a single dose of caudal ropivacaine with 1 MAC of sevoflurane in children aged 1–6 years old, and the MLAC of caudal ropivacaine was 0.066%. Deng et al. [5, 11] reported that the MLAC of caudal ropivacaine with 0.5 MAC enflurane was 0.11% in children aged 1–5 years old, and it was 0.107% in preschool age children with 0.7 MAC sevoflurane. The MLAC values of ropivacaine were 0.128% in the present study, and the value reported in our study was consistent with the value reported elsewhere, but it was different from certain other studies. This discrepancy could be mainly attributed to individual factors, the concentration and volume of caudal injection, the use and concentration of inhaled or intravenous anesthetics, methods of measuring MLAC value [9, 10]. Sevoflurane have a much stronger inhibitory effect compared with propofol within concentrations close to their reported 50% effective concentrations [12, 13]. And sevoflurane strongly decrease the required concentrations of local anesthetics compared with midazolam or propofol. Also, MLAC estimates could be influenced by starting concentration, number of crossovers, increment size of concentration when using up-down method for analysis of quantal data [10].

Lots of previous studies had performed to evaluate the effect of different doses of ketamine on the post-operative analgesia. However, to the best of our knowledge, no Chinese or English studies have been designed to evaluate the effect of different dose of caudal ketamine on MLAC of ropivacaine. Our results showed that caudal ketamine did not influence the MLAC of ropivacaine for intra-operative caudal analgesia in young children. However, it prolonged the duration of postoperative analgesia in this study. Our results were consistent with previous studies of caudal ketamine in association with local anesthetics for post-operative analgesia [14–17]. Our results showed that caudal ketamine prolonged the duration of post-operative analgesia in a dose-dependent manner. Caudal ketamine with 0.25% bupivacaine was used in doses of 0.25, 0.5, and 1 mg/kg, obtaining mean postoperative analgesia durations of 7.9, 11, and 16.5 h, respectively [18]. In another study, the mean durations of caudal analgesia in caudal 0.25% bupivacaine with 0.25, 0.5, 1 mg/kg ketamine were 8.8, 22.1, and 25.2 h, respectively [19]. Adequate postoperative analgesia has also been achieved by combining caudal ropivacaine with ketamine. Adding 0.25 mg/kg ketamine to 0.2% ropivacaine provided analgesia up to 9 h in Lee et al. study [14]. Adding 0.5 mg/kg ketamine to 0.2% ropivacaine produced analgesia that extended about 11–17 h [15–17].

Caudal ketamine provide analgesia of central nervous system effects by both a direct effect on the spinal cord and a systemic absorption effect. Ketamine exerts its analgesic and anesthetic effects by binding to a subset of glutamate receptors influenced by the agonist N-methyl-D-asparate (NMDA). Caudal ketamine produced analgesic effects by acting on NMDA receptors in the lumbar spinal cord, or from μ-opioid receptors agonist activity and interaction with voltage-sensitive sodium channels [20, 21]. Koinig et al. [22] showed that the mean time to maximum ketamine plasma concentration after caudal ketamine administration was about 20 min and the maximum ketamine plasma concentrations after caudal injection were significantly lower than that after intramuscular administration of ketamine. Caudal ketamine alone was typically used to provide post-operative analgesia in children in several studies [23, 24]. These suggested that caudal ketamine analgesic effect was provided primarily by a direct effect on the spinal cord. In our study, skin incision was performed at 20 min after caudal injection. And at this time, it was the peak of ketamine plasma concentration, may not be the peak of ketamine concentration in the spinal cord nervous after caudal ketamine injection. It’s the limitation in our study and future studies need to confirm this.

Ketamine is a low molecular weight and relatively high lipid soluble drug, and this characteristic of ketamine leads to release slowly from the lipid composition of the spinal cord [25]. Elimination half-life after epidurally administered ketamine was two times longer than that after intravenous ketamine [26]. Hence, the synergistic effect between ketamine and ropivacaine primarily resulted in the prolongation of post-operative caudal analgesia, but not for intra-operative caudal analgesia. The doses of caudal ketamine were from 0 to 1 mg/kg in the present study. This study’s limitation is that we’re not sure whether ketamine in larger doses than 1 mg/kg could decrease the MLAC of ropivacaine for intra-operative caudal analgesia in children. However, the range of doses in our study is representative of commonly used doses in clinical medicine.

## Conclusions

In summary, this study indicated that the addition of caudal ketamine ranged from 0 to 1 mg/kg to ropivacaine prolonged the duration of post-operative analgesia; however, it did not decrease the MLAC of caudal ropivacaine for intra-operative analgesia in children.

## Data Availability

The datasets are not publicly available, but available from the corresponding author on reasonable request.

## Abbreviations

FLACC: Face, Legs, Activity, Cry, Consoling” tool
PONV: Postoperative Nausea and Vomiting
MLAC: Minimum Local Anesthetic Concentration
ASA: American Society of Anesthesiology
NMDA: N-methyl-D-asparate
MAC: Minimal Alveolar Concentration
